# Comparative analysis of plant use in peri-urban domestic gardens of the Limpopo Province, South Africa

**DOI:** 10.1186/1746-4269-10-35

**Published:** 2014-04-04

**Authors:** Gabolwelwe KE Mosina, Alfred Maroyi, Martin J Potgieter

**Affiliations:** 1 Department of Microbiology and Plant Pathology; Forestry, Agriculture and Biotechnology Institute, University of Pretoria, Lynnwood Road, Alice, South Africa; 2 Medicinal Plants and Economic Development (MPED) Research Center; Department of Botany, University of Fort Hare, Private Bag X1314, Alice 5700, South Africa; 3 Department of Biodiversity, School of Molecular and Life Sciences, University of Limpopo, Private Bag X1106, Sovenga 0727, South Africa

**Keywords:** Cultivated plants, Limpopo Province, Livelihoods, Peri-urban, Urbanisation, Useful plants

## Abstract

**Background:**

Relatively little has been researched or published on the importance of peri-urban domestic gardens as part of a household livelihood strategy in South Africa. Due to lack of comprehensive data on peri-urban domestic gardens, their potential value as luxury green space, provision of food, income and ecosystem services to the fast growing urban population in South Africa is not clearly known. The aim of this study was to document differences and similarities in plant use and diversity in domestic gardens of two peri-urban communities in the Limpopo Province that differ in proximity to an urban area.

**Methods:**

Data on plant use categories of 62 domestic gardens in the peri-urban areas of the Limpopo Province were collected in Seshego and Lebowakgomo. Semi-structured interviews, observation and guided field walks with 62 participants were employed between May and October 2012.

**Results:**

A total of 126 plant species were recorded for both Seshego and Lebowakgomo. Domestic gardens in the more remote areas of Lebowakgomo were characterized by higher percentage of food plants (47 species, 83.8% of the total food plants recorded) and medicinal plants (31 species, 83.7%). Lebowakgomo domestic gardens were also characterized by higher numbers of indigenous plants (76.7%) showing similarities to the natural surrounding vegetation in terms of plant species. On the contrary, domestic gardens of Seshego on the periphery of the city centre were characterized by higher percentage of exotic species (81.8%) and ornamental plants (73%), with food plants playing a supplementary role. Comparison of the two areas demonstrated a remarkable difference in plant use and composition.

**Conclusions:**

This study revealed that there are differences in utilization of plant resources between households on the edge of an urban centre and those in the more remote areas. Food and medicinal plants play an important role in remote areas; while ornamental plants play an important role in urban domestic gardens. But the collective desire for food, medicinal and ornamental plants by both communities on the edge of an urban centre and those in the more remote areas highlight the importance of plant resources in domestic gardens.

## Background

There is growing interest in the documentation of goods and services provided by home gardens throughout the world [1,2], including South Africa [3-7]. The Millennium Ecosystem Assessment [8] defined goods and services as the benefits that humans obtain from natural or semi-natural ecosystems. Fernandes and Nair [1] defined a home garden as an intensively worked land-use system involving deliberate management of multipurpose plants in association with agricultural crops and invariably livestock, within the compounds of individual households. Researchers suggest that, besides the provisioning of food, fuel and medicines; home gardens also provide cultural, regulating services and serve as a habitat to other organisms. Based on studies conducted in the North West Province, South Africa, Molebatsi et al. [4], defined a domestic garden as a luxury space around the house used for relaxation, play areas, keeping pets, outdoor eating and cultivation of ornamental plants. Urban domestic gardens provide multiple ecosystem services that contribute to quality of life in cities, air quality regulation, carbon capturing [9], temperature regulation [10], storm water run-off mitigation [11], as well as recreational benefits and social cohesion [12]. Kuruneri-Chitepo and Shackleton [13] showed that urban biodiversity enables urban inhabitants to interact with nature, thereby enhancing appreciation and understanding of the important ecological, social and psychological functions green areas perform.

The main difference between urban and rural gardens lies in the purpose and use of the gardens, resulting in different species grown and maintained in these gardens. A rural home garden is regarded as part of a household livelihood strategy, a natural asset through which sustainable use of resources, particularly for the livelihoods of the poor are achieved [14]. Nair [15] showed that high number of ornamental plants in urban gardens is associated with the aesthetic role of domestic gardens in cities, since they are not used for subsistence, except among low income city dwellers. Similarly, Reichard and White [16] showed that large number of plant species introduced into the urban environment are for horticultural purposes. Relatively little has been researched or published on the importance of peri-urban domestic gardens in South Africa. Due to lack of comprehensive data on peri-urban domestic gardens, their potential value as luxury green space, provision of food, income and ecosystem services to the fast growing urban population in South Africa is not clearly known. For the purpose of this study, a home garden is defined as an area adjacent to a household dwelling, where the household has control over the area characterized by a diversity of organisms, hereafter referred to as the domestic garden.

The peri-urban areas are formerly “rural” localities that are now due to the rapid expansion of South Africa’s metros and major towns lie outside the urban edge [17]. The wealth gap between rich and poor in South Africa is most visible on the urban outskirts. Apartheid spatial planning policies was racially-based and segregated black populations in areas some distance from urban cores, while whites resided in suburbs typical of any city in the first world [18]. As a result of this racially-based ideology, South Africa’s urban peripheries are usually occupied by the poor [17]. Most informal settlements evolve on the urban edge and low cost, subsidised housing developments to improve the lives of the poor tend to be located in the same areas [19]. The same author found that the peri-urban poor lead multifaceted livelihoods characterized by small scale farming which contributes to household’s income and nutritional needs. This study was therefore undertaken within the wider problem of understanding the differences and similarities in plant use and diversity in two peri-urban communities in the Limpopo Province of South Africa that differ in proximity to an urban area. Our hypothesis states that food production in domestic gardens is important for households in remote areas and becomes less important for households on the edge of an urban centre. We tested this hypothesis by compiling a list of plant use categories, assessed their importance in domestic gardens and how indigenous and non-native plants were used by both households on the edge of an urban centre and those in the more remote areas.

## Materials and methods

### Study area

**Figure 1:**
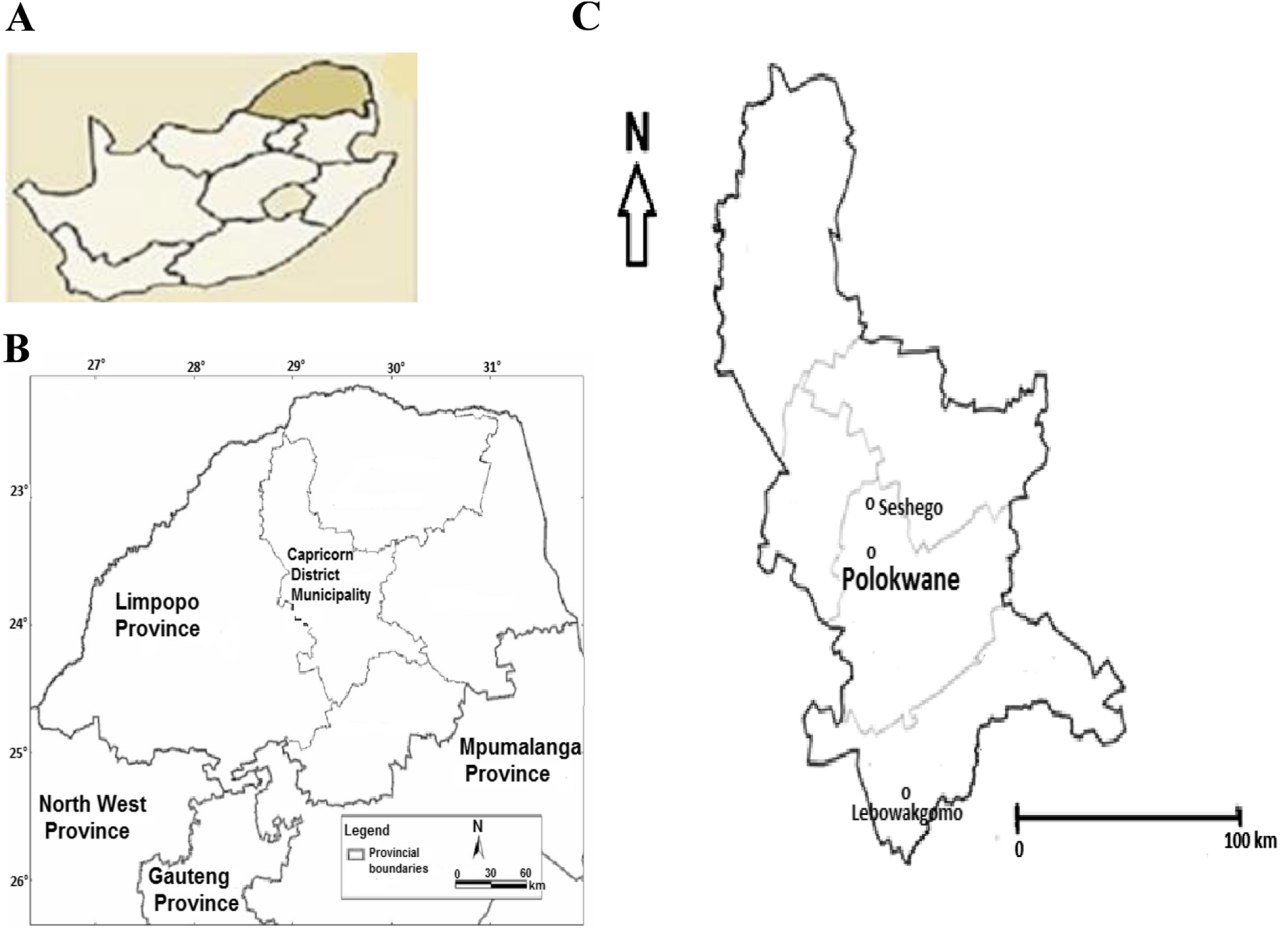
**Geographical location of the study area. A**: Map of South Africa, illustrating the geographical position of the study area. **B**: Map of Limpopo Province, showing the geographical position of the Capricorn District. **C**: Detailed map of the study area.

The study was conducted in Seshego (23°15'S29°23'E) and Lebowakgomo (24°31'S29°57'E) (Figure 1), two peri-urban areas in the Capricorn District, Limpopo Province. Limpopo Province is a predominantly rural province with 86.7% of its population living in the rural areas in 2008 [20]. Most urban settlements in the province are informal and there are no metropolitan centres. A metropolitan centre is a municipality that execute all the functions of local government for a city, and that have sufficient resources to perform municipal function [21]. The rural character of the province further highlights that Limpopo Province is not an industrialized province. The province’s source of economic activities are centred principally around agriculture and mining [22]. The province has the second highest estimated net migration after the Eastern Cape Province [23]. The migration trend can be attributed mainly to the rural character of the province, which has led to economically active people migrating either to Polokwane municipality or the more industrialized neighbouring province of Gauteng [22]. Polokwane municipality is located at the centre of the Limpopo Province and it is situated at the core of the province’s economic development. Polokwane is developing at a rapid pace and the mining industry has boomed over the last decade. Situated on the outskirts of Polokwane municipality are Seshego and Lebowakgomo, which are less formal settlements experiencing enormous influx from rural to urban migration trends. Seshego is located 13 km north of Polokwane and the township was planned as a dormitory town for workers in Polokwane city [24]. Seshego is nearest to the economic core of Polokwane and thus has the best access to the formal economy of the city. Lebowakgomo is located 55 km south of Polokwane. The main employment sector in Lebowakgomo is the mining industry. These areas are in dire need of upgraded physical infrastructure, services and economic prospects of employment to cope with the informal influx of more people who want access to an improved quality and standard of living.

The studied areas are semi-arid, susceptible to frequent droughts and characterized by summer rainfall. Mean annual rainfall ranges from 300 to 500 mm [25]. Daily temperatures vary from mid-20°C to mid-30°C, with an average range of between 17°C and 27°C in summer and 4°C to 20°C in winter [26]. According to the vegetation classification of Mucina and Rutherford [27], the study areas have a semi-arid savanna, characterized by a mixture of trees, shrubs and grasses. Dominant tree species include *Acacia* spp., *Albizia* spp., *Combretum* spp. and *Sclerocarya birrea*, with patches of *Hyparrhenia* spp., *Eragrostis* spp., *Heteropogon* spp. and *Digitaria* spp. grasses.

### Data collection

Data on plant use categories in urban domestic gardens of Seshego and Lebowakgomo were collected by means of semi-structured and structured interviews and personal observation. Thirty one randomly selected individuals from each study area were interviewed between May and October 2012 (Table 1). Table 1 shows the demographic characteristics of the participants. Of the sixty two participants, 69.4% were female and 30.6% were male. Their ages ranged from 19 to 73 years, with 48 years as the median. The majority of participants were married (58.1%), 32.3% divorced and 9.7% single (Table 1). The majority of households (80.7%) comprised between three and six family members, while 17.7% lived alone and one household had seven family members (Table 1). The majority (53.2%) of the participants were educated up to secondary level, while 29% had attained tertiary education, 11.3% had attained primary level and 6.5% were illiterate. More than half of the participants (56.5%) were unemployed, surviving on less than R2000 (US$200) a month (Table 1). A very small proportion of the participants had constant income as either self-employed (14.5%) or employed by a company (29%) and 27.4% of the participants did not disclose their monthly income (Table 1).

**Table 1:** Socio-economic characteristics of the study sample, N = 62

**Socio-economic variables**		**Number**	**%**
**Gender**	Female	43	30.6
	Male	19	69.4
**Age (years)**	<20	1	1.6
	20-29	11	17.7
	30-39	10	16.1
	40-49	17	27.4
	50-59	11	17.7
	>60	12	19.4
**Marital status**	Single	6	9.7
	Married	36	58.1
	Divorced	20	32.3
**Household size**	1-2	11	17.7
	3-4	28	45.2
	5-6	22	35.5
	7-8	1	1.6
**Highest level of education**	No education	4	6.5
	Primary	7	11.3
	Secondary	33	53.2
	Tertiary	18	29.0
**Occupation**	Unemployed	35	56.5
	Employed	18	29.0
	Self-employed	9	14.5
**Combined monthly income**	Less than R1000*	22	35.5
	R1001-2000	7	11.3
	R2001-3000	7	11.3
	R3001-4000	4	6.5
	R4001-5000	2	3.2
	More than R5001	3	4.8
	Not disclosed	17	27.4

*1 Rand = US$0.1.

Verbal informal consent was obtained from each individual who participated in the study, and the researchers adhered to the ethical guidelines of the International Society of Ethnobiology (http://www.ethnobiology.net). The aim and purpose of the investigation was explained to selected participants. The questionnaire used during interviews was designed to gather data on socio-economic characteristics of the participants and useful plant species (food, medicinal and ornamental) grown and maintained in the domestic gardens. A plant species was included in this study if the domestic garden owner could indicate its use. Voucher specimens of plants identified in domestic gardens were collected during the field trips when encountered for the first time and again when they were flowering or fruiting, for easy identification. The voucher specimens were processed using standard taxonomic procedures [28,29]. Each herbarium specimen included important parts such as leaves, stems, flowers and fruits whenever available. For small herbaceous plants, the whole plants were collected. These specimens were deposited for future reference at the Larry Leach Herbarium (UNIN) of the University of Limpopo.

### Data management and analysis

The data collected were entered in Microsoft Excel 2007 programme and were later analyzed for descriptive statistical patterns. During analysis, data on useful plants as provided by the participants were summarized into major themes by content analysis [30]. Through content analysis, it was possible to distil words into fewer content-related categories, sharing the meaning [31]. Inconsistencies and unique statements were noted and given particular attention. Descriptive statistics, such as percentages and frequencies were used to analyse the data obtained from the questionnaires. Bar graphs were generated using Microsoft Excel 2007 programme.

Species are described as native or alien based on Pyšek et al. [32]. According to Pyšek et al*.*[32], naturalized species are defined as aliens that reproduce consistently without direct human intervention, and invasive aliens as naturalized species producing offspring in large numbers and at considerable distances from the parent plants with the potential to spread over a large area. This definition of invasive alien species used in this study is different from the Convention on Biological Diversity (CBD) Conference of Parties’ definition of an invasive alien species, where an alien is defined as a species outside its indigenous geographic range, whose introduction and spread threatens biodiversity [33]. Another important species classification used in this study is the “indigenous cultivated” category, referring to species indigenous to South Africa and not occurring naturally in Seshego or Lebowakgomo, Limpopo Province, but cultivated in domestic gardens. The origin of “indigenous cultivated” species was determined from Germishuizen et al. [34]. In this study, the term “indigenous” is used to refer to a plant species that is naturally occurring and usually not cultivated in Seshego or Lebowakgomo, Limpopo Province, South Africa. Exotic is a plant species in Seshego or Lebowakgomo, Limpopo Province, whose presence there is due to intentional or unintentional human involvement or which has arrived there without the help of people from an area in which they are indigenous [32]. A weedy species is defined as a plant (not necessarily exotic) that grow in sites where it is not wanted and which has detectable economic or environmental impact or both [32].

## Results and discussion

### Plant diversity

**Figure 2:**
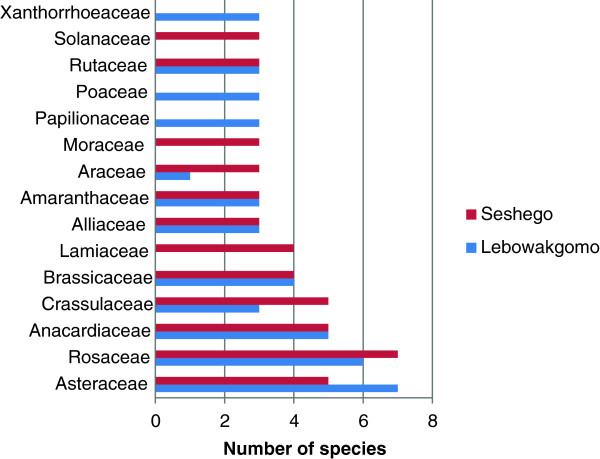
Families with the largest number of plants grown and maintained in domestic gardens of Lebowakgomo and Seshego in the Limpopo Province.

A total of 126 plant species used by both Seshego and Lebowakgomo residents was compiled. Lebowakgomo domestic gardens had higher species numbers (94 species, 74.6% of the total) when compared to Seshego (84 species, 66.7% of the total) (Table 2). Lebowakgomo was also characterized by more plant families and genera than Seshego (Table 2). Asteraceae, Anacardiaceae and Rosaceae had at least five species in both Lebowakgomo and Seshego (Figure 2). Asteraceae, Anacardiaceae and Rosaceae are among the largest families in South Africa characterized by more than 100 species each [34]. Families Papilionaceae, Poaceae and Xanthorrhoeaceae were recorded in Lebowakgomo only, while Lamiaceae, Moraceae and Solanaceae were recorded in Seshego only (Figure 2). Seshego domestic gardens were characterized by higher number of exotic species, 81.8% of the total exotic species recorded in the study, while Lebowakgomo domestic gardens had higher proportion of indigenous species (76.7% of the total indigenous species recorded in the study) (Table 2). Some of the indigenous species recorded in Lebowakgomo domestic gardens only were *Erythrina lysistemon* Hutch., *Vigna unguiculata* (L.) Walp. and *Vigna subterranea* (L.) Verdc. (Papilionaceae family), *Aloe ecklonis* Salm-Dyck and *Aloe* sp. (Xanthorrhoeaceae). Among the exotic species recorded in Seshego domestic gardens only were *Lavandula angustifolia* Mill. and *Rosmarinus officinalis* L. (members of Lamiaceae family), *Ficus carica* L. and *Morus alba* L. (Moraceae) and *Capsicum frutescens* L. and *Lycopersicon esculentum* L. (Solanaceae). Exotic species appear to dominate in the urbanized Seshego while in Lebowakgomo which lies on the outskirts of Polokwane city, the indigenous plant species showing similarities in terms of plant species to the natural vegetation of the area dominates. The dominance of exotic species in Seshego is mainly due to deliberate introductions of exotic species in domestic gardens. These results correlate strongly with previous research by McKinney [35] which showed that urbanization reduces the diversity and abundance of indigenous species, because of the homogenisation of the habitat in urban areas.

**Table 2:** Summary of plant species grown and maintained in domestic gardens of Lebowakgomo and Seshego in the Limpopo Province

		**Seshego (%)**	**Lebowakgomo (%)**
**Taxonomic rank**	Family	45 (72.6%)	52 (78.8%)
	Genera	77 (70%)	82 (74.5%)
	Species	84 (66.7%)	94 (74.6%)
**Origin**	Exotic	54 (81.8%)	48 (72.7%)
	Indigenous	30 (50%)	46 (76.7%)
	Cultivated indigenous	5 (41.7%)	9 (75%)

Lebowakgomo domestic gardens had higher number of “cultivated indigenous” species (75% of the total), against 41.7% recorded in Seshego domestic gardens (Table 2). Among these were the following species, introduced to Lebowakgomo domestic gardens from other provinces of South Africa: *Agapanthus africanus* ssp *africanus* (Western Cape), *Clivia miniata* var. *miniata* (Eastern Cape, KwaZulu-Natal, Mpumalanga), *Dietes grandiflora* (Eastern Cape, KwaZulu-Natal), *Drimiopsis maculata* (Eastern Cape, Gauteng, KwaZulu- Natal, Mpumalanga), *Euryops chrysanthemoides* (Eastern Cape, KwaZulu-Natal), *Haworthia fasciata* (Eastern Cape), *Pelargonium peltatum* (Eastern Cape, Western Cape ), *Pelargonium zonale* (Eastern Cape, KwaZulu-Natal, Western Cape), *Strelitzia nicolai* (Eastern Cape, KwaZulu-Natal) and *Strelitzia reginae* ssp *reginae* (Eastern Cape, KwaZulu-Natal). *Begonia homonyma* (Eastern Cape, KwaZulu-Natal), *Haworthia fasciata*, *Lobostemon fruticosus* (Western Cape), *Pelargonium peltatum* and *Pelargonium zonale* were introduced to Seshego domestic gardens. The number of “cultivated indigenous” species is higher in Lebowakgomo domestic gardens than in Seshego probably because households get these plants from two nurseries in Lebowakgomo, where “indigenous cultivated” species are readily available. Lubbe et al. [36] argued that home gardens in the Tlokwe municipality, North West Province, South Africa, have high number of ornamental plants because home gardeners get these plants from nurseries in the city and also nurseries are promoting planting of ornamental plants. The presence of these species from other provinces may point out to the possibility of exchange and sharing of ethnobotanical information on these species. Previous research by Gilmore [37] showed that the relations of people to their useful plants and that of other regions near or further away aids in measuring their cultural status and their contacts with each other. Moreover, households give home garden products to neighbours and relatives, and this exchange between households and relatives strengthen relationships [38].

### Plant uses

**Figure 3:**
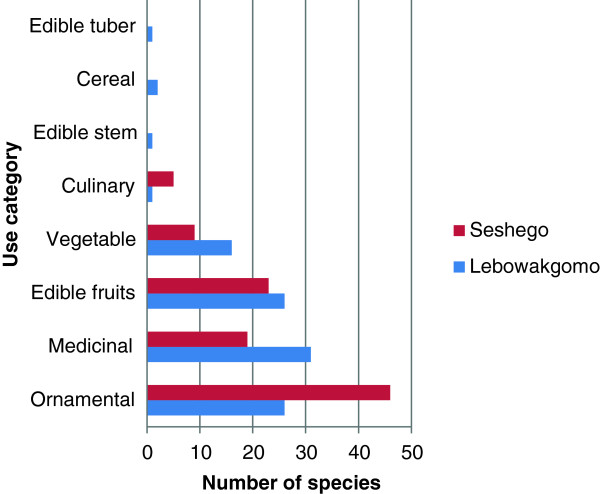
Number of plants used for food, medicine and as ornamentals in the Limpopo Province.

Eight major uses of domestic garden plants identified in this study (Figure 3) were: cereal, culinary herb, edible fruit, edible stem, edible tuber, medicinal, ornamental and vegetable. The dominant plant use category in Seshego domestic gardens was ornamental with 46 species (73% of total ornamentals recorded) (Figure 3), followed by edible fruits (23 species, 79.3%), medicinal (19 species, 51.4%), vegetable (9 species, 50%) and culinary herbs (5 species, 83.3%). Important ornamental plants (grown and maintained by more than 50% of the participants) included *Catharanthus roseus* (L.) G. Don. (also used as medicine, *Cyperus sexangularis* Nees, *Euryops chrysanthemoides* (DC.) B. Nord. and *Tecoma stans* (L.) Juss. ex Kunth. Fruit trees grown and maintained by at least 50% of the participants included *Carica papaya* L. (also used as medicine), *Citrus limon* (L.) Burm. f., *Citrus sinensis* (L.) Osbeck, *Mangifera indica* L., *Musa* sp*., Persea americana* Mill., *Prunus persica* (L.) Stokes and *Sclerocarya birrea* (A. Rich.) Hochst. ssp *caffra* (Sond.) Kokwaro (also used as medicine). These results corroborate results obtained by Nair [15] and Eichemberg et al. [39], which showed high number of ornamental plants in urban gardens. Eichemberg et al. [39] assessed species composition in urban home gardens in Rio Claro municipality, southeast Brazil and documenting three major plant use categories, dominated by ornamentals (63%), alimentary (24%) and medicinal (23%). Nair [15] and Eichemberg et al. [39] argued that ornamental plants are associated with the aesthetic role of domestic gardens in cities, since they are not used for subsistence, except among low income city dwellers. Research by Eichemberg et al. [39] showed that the number of ornamental plants has increased in urban areas in response to the process of modernization and the large supply of these plants in cities. Previous research in the Midlands Province, Zimbabwe [14], showed vegetable production (20%) to be a major home garden activity in rural areas, followed by edible fruits production; ornamentals, hedging and shade plants; building, timber, firewood and construction material (18% each) and medicinal plants (11%). Similar results were obtained in the assessment of rural home gardens in Zvishavane District, Zimbabwe [38] where vegetable production (34.2%) dominated, followed by edible fruits production (30.1%), ornamentals (20.5%) and medicinal plants (13.7%).

The dominant plant use category in Lebowakgomo domestic gardens was medicinal with 31 species (83.8% of the total herbal medicines recorded) (Figure 3), followed by edible fruits (26 species, 89.7%) and ornamentals (26 species, 41.3%), vegetable (16 species, 88.9%), cereal (2 species), edible stem and tubers (1 species each). The total number of plants with edible parts (47 species, 83.8% of the total food plants recorded) surpasses those used as herbal medicines, therefore, domestic gardens in Lebowakgomo play an important role in food production. Moreover, only a few species are cultivated as food plants, with over 50% of the daily global carbohydrate and protein needs derived from three crops: maize, wheat and rice [40]. Traditional vegetables often associated with rural domestic gardens recorded in Lebowakgomo included *Brassica juncea* (L.) Czern., *Citrillus lanatus* (Thunb.) Matsum. & Nakai, *Cleome gynandra* L., *Corchorus olitorius* L. var. *olitorius, Cucurbita pepo* L., *Ipomoea batatas* (L.) Lam., *Spinacia oleracea* L., *Vigna subterranea* and *Vigna unguiculata*. Other plants often associated with rural agroecosystems cultivated in Lebowakgomo were *Sorghum bicolor* (L.) Moench (Sorghum), *Saccharum officinarum* (sugar cane) and *Zea mays* (maize). *Saccharum officinarum* was grown in patches in damp places at low elevation for its edible stem, *Sorghum bicolor* was cultivated as a cereal and *Zea mays* was grown as a cereal and for its green mealies roasted or cooked. Indigenous fruit trees cultivated and/or maintained by households in Lebowakgomo domestic gardens included *Harpephyllum caffrum* Bernh., *Sclerocarya birrea* ssp *caffra* and *Vangueria infausta* Burch. ssp *infausta.* The results of this study provides additional support to the general assertion that domestic gardens in remote and rural areas are used for production of fruit, vegetable, medicinal and ornamental plants [41].

### Cultivation of weedy species in domestic gardens

About 10% (13 species) of the total garden flora recorded in this study are declared weeds and invaders in South Africa, listed under the Conservation of Agricultural Resources Act (1983) No. 43 of 1983. Among these were: *Agave americana* L. (medicinal), *Catharanthus roseus* (medicinal/ornamental), *Duranta erecta*, *Echinopsis spachiana* (Lem.) Friedrich & G.D. Rowley (medicinal/ornamental), *Eriobotrya japonica* (Thunb.) Lindl. (edible fruit), *Morus alba* (edible fruit), *Nasturtium officinale* W.T. Aiton (ornamental), *Nephrolepis exltata* (L.) Schott (Ornamental), *Opuntia ficus-indica* (L.) Mill. (edible fruit/ornamental), *Passiflora edulis* Sims (edible fruit), *Psidium guajava* L. (edible fruit), *Schinus terebinthifolius* Raddi (medicinal/ornamental) and *Tecoma stans* (ornamental). The majority of these species pose an immediate and significant threat by virtue of their aggressive qualities and having the capacity to invade natural habitats and overwhelm some of the indigenous species [42]. Second to habitat destruction and modification, alien invasion is recognized as having the largest impact on natural vegetation, ecosystem processes and interfering with agricultural practices [43-45]. Studies by Bigirimana et al. [44,45], Maroyi [46] and Semenya et al. [47] showed that invasive plants may also have positive economical, social and ecological significance and these need to be taken into account when assessing the costs resulting from invasions. As part of this management strategy, domestic garden owners should be educated on the management of some of the invasive species, especially those listed in category 1 of the Conservation of Agricultural Resources Act (1983) No. 43 of 1983 [42].

## Conclusion

This study revealed that there are differences in utilization of plant resources between households on the edge of an urban centre and those in the more remote areas. Domestic gardens in the more remote areas, the city outskirts were characterized by higher percentage of food and medicinal plants. The more remote areas were also characterized by higher numbers of indigenous plants showing similarities to the natural surrounding vegetation. On the contrary, domestic gardens on the periphery of the city centre were characterized by higher percentage of exotic species and ornamental plants, with food plants playing a supplementary role. The hypothesis that food production in domestic gardens becomes less important along a rural to urban gradient is supported. Comparison of the two areas demonstrated a remarkable difference in plant use, including significant differences in plant composition in domestic gardens. The collective desire for food, medicinal and ornamental plants by both communities in Lebowakgomo and Seshego highlight the importance of plant resources in domestic gardens. Future researchers could use some of these differences in plant diversity and usage along a rural to urban gradient to study multifaceted characteristics associated with ethnobotanical knowledge in urban and metropolitan centres. This is particularly important as natural environment and native biodiversity are declining in urban areas due to urbanisation and human development. There is a need to show how under different conditions the importance of biodiversity in domestic gardens varies, and how this is related to environmental degradation and food ecology.

## Competing interests

The author declares that they have no competing interests.

## Authors’ contributions

GKEM wrote the manuscript, AM and MJP, respectively helped to finalize the manuscript. Field work was carried out by GKEM and AM. All the authors read and approved the final manuscript.
